# Metamemory: Metacognitive Strategies for Improved Memory Operations and the Role of VR and Mobiles

**DOI:** 10.3390/bs12110450

**Published:** 2022-11-14

**Authors:** Athanasios Drigas, Eleni Mitsea, Charalabos Skianis

**Affiliations:** 1 Net Media Lab & Mind & Brain R&D, N.C.S.R. ‘Demokritos’, 15341 Agia Paraskevi, Greece; 2 Communication Systems Engineering Department, University of the Aegean, 811 00 Mitilini, Greece

**Keywords:** metamemory model, metacognitive strategies, mnemonics, smartphones, virtual reality

## Abstract

Memory is one of the most vital cognitive functions, affecting almost all aspects of human life. Meta-memory is considered a special part of metacognition that enables humans to acquire mnemonic knowledge and meta-skills to take control of their memory functions. In the digital era, the use of mobile applications to improve memory is constantly gaining ground, while virtual reality is considered a promising technology for memory rehabilitation. The current study aimed to present a metamemory framework based on eight fundamental principles of metacognition. The theoretical model is complemented by a set of meta-mnemonic strategies while emphasizing the role of virtual reality and mobile applications in metamemory skills training. The metamemory strategies framework supported by virtual reality and mobile applications provides a training paradigm for implementation in general, special, and vocational education.

## 1. Introduction

Memory is one of the most vital cognitive functions, affecting almost all aspects of human life. A strong, active memory is the foundation of our whole mental functioning, ensuring survival, learning, and achievement. Even self-identity is dependent on our memories. Despite being studied as thoroughly as a few other cognitive functions, we still know very little about memory’s underlying nature [1].

Recent evidence suggests that memory problems have dramatically increased even in the healthy population. For instance, recent studies have already shown that people who have experienced COVID-19 infection struggle with long-lasting memory problems [2]. Memory problems are apparent among the most serious diseases plaguing the modern world, including Alzheimer’s, dementia, Parkinson’s, depression, obsessive–compulsive disorder, anxiety, and various learning disorders. Recent studies have revealed that the number of people living with dementia approximately doubles every five years [3,4].

Memory problems are considered one of the most common manifestations of children with learning difficulties. Previous studies have reported that memory problems are one of the central reasons for academic underachievement. Students with memory problems face difficulties in manipulating complex information, recalling events and facts, reading problems, recognizing visual symbols, understanding concepts and ideas, and solving problems. Most importantly, memory problems are closely associated with reduced awareness and control over knowledge. It is common to notice students’ inability to follow instructions, strategically use previous knowledge to acquire new knowledge, apply the acquired knowledge in different contexts, learn from mistakes, and be flexible in altering strategies when needed [5].

At the same time, researchers are investigating the reasons why some people have exceptional memory. For instance, some people can recall limitless information without effort [6]. Parker et al. [6] examined the case of a woman who was able to effortlessly recall specific events with remarkable precision and reliability dating back to the age of 18 months. However, the neuropsychological tests revealed that beyond exceptional memory, the woman had difficulty with tasks that required her to control her memories and formulate retrieval strategies. Ericsson et al. [7] examined the superior memory capacity of memoirists. After extensive evaluation, the results indicated that famous memorists’ abilities are mediated by encoding strategies acquired after thousands of hours of practice.

A growing body of literature reveals that memory can be improved through targeted training [8]. Other studies outline that metacognitive strategies are closely associated with better control over memory functions [9,10]. Mnemonics refer to a large group of strategies that intend to make memorization easier [11]. However, there are unanswered questions regarding the human ability to control memory processes and the training strategies which can help learners to take control of their memory abilities.

Metamemory can be defined as the awareness and control people have over their memory operations. Metamemory is closely linked with a set of meta-abilities including the ability to gather information through real-time monitoring about the current state of the memory system. The metamemory concept has been closely linked with the theory of metacognition [12].

Metacognition refers to a set of regulatory meta-abilities and meta-skills that are intentionally employed aiming at the smooth operation of the cognitive and psychophysiological system as a means of gaining functional capability, self-efficacy, independent living, and life satisfaction. Metacognition engages consciousness-raising skills and strategies including individuals’ ability to monitor, regulate, and adapt their internal mental processes, recognize the difference between functional and dysfunctional states of mind, and consciously choose those states that awaken the full range of their abilities and identity [13,14].

Assistive technology, including virtual reality (VR) and mobile applications, has already gained interest as innovative interventions in a wide range of clinical settings [15,16,17,18,19,20]. VR is defined as a technology that induces virtual immersion in a digital environment through the use of a computerized visual simulation, allowing users to be engaged in an interactive three-dimensional environment rich in sensory and emotional experiences [21]. Virtual reality is a cutting-edge technology that is a powerful tool for the assessment and rehabilitation of cognitive functions. Researchers argue that virtual reality may provide new approaches to the treatment of memory impairments [22,23]. Mobile health is increasingly gaining ground and devices are now widely accessible to the average citizen. Because of the time-consuming nature of memory training, the development of mobile solutions for memory training is now a necessity [24,25,26,27,28].

This study aimed to present a metamemory framework based on metacognition principles. Intending to provide a functional metamemory model, we support the theoretical framework with a wide range of evidence-based strategies. Special emphasis was given to the role of assistive technologies, namely mobile technologies, and virtual reality. The proposed metamemory framework intends to renew the debate about the concept of metamemory, develop a new theoretical framework bridging two interrelated concepts, namely metamemory and metacognition, and provide implications for practice and training. According to our knowledge, this is the first metamemory strategies framework that has been developed in terms of metacognition.

## 2. Methodology

The current theoretical article aimed to renew the debate about the concept of metamemory, redefine the role of metacognition in memory control processes, and highlight the importance of metacognitive strategies for developing metamemory skills with the assistance of virtual reality and mobile technology.

The first section of the study focuses on the foundation of a metamemory model based on metacognition. We started by looking for well-recognized theories and models of memory and metamemory, which supported the foundation of the proposed metamemory model. In addition to well-known theories, another criterion for their selection was whether they directly or indirectly recognize the occurrence of memory control processes or the importance of employing strategies to regulate memory functions. The presentation of important factors as well as lifestyle variables that influence memory functions including the meta-ability to manage memory operations was also emphasized.

The second part of the article, which essentially complements the theoretical model, presents metacognitive strategies for memory control training. The strategy presentation was divided into two axes. The first deals with strategies and techniques, while the second examines the effectiveness of assistive technologies in metamemory training. Specifically, we investigated the effectiveness of virtual reality and mobiles as assistive tools in training metamemory skills. The strategies section constitutes a review of evidence-based studies. There were no strict age criteria in the selection of the articles, as the aim of the research was to determine whether digital technologies can support metamemory training. The selection of the articles was mainly based on the compatibility of the strategies with the proposed metamemory model. As a result, emphasis was placed on studies that offered metacognitive techniques for training awareness and memory control abilities. The studies on new technologies were chosen using the same criteria. The articles were chosen for their emphasis on the effectiveness of virtual reality and mobiles on metamemory skills development. Priority was given to studies combining technology with mnemonic methods. However, studies evaluating the usefulness of virtual reality and mobile technologies in assisting memory control processes were also included.

## 3. Theoretical Background

### 3.1. The Transition from Memory to Metamemory Theories

Atkinson and Shiffrin [29], first, proposed a theoretical framework, according to which memory consists of three distinct store systems: the sensory register, the short-term store, and the long-term store. As they stated, the stimuli received from the environment pass through three stages (encoding, storage, and retrieval) [30] where the transition from one phase to the next is inextricably tied to control mechanisms. Unless control processes intervene, the information is doomed to be lost. For this purpose, individuals must utilize strategies to exercise voluntary control over their memory processes.

Atkinson et al. [29] presented short-term memory as a unitary system with limited storage and processing capacity. Baddeley and Hitch [31] counter-proposed a working memory model as a powerful engine in human information processing. Instead of a unitary system with limited processing, they described working memory as a three-component system that can retain and process a limited amount of information. This model includes a control system with limited attentional capacity, called the central executive, which is supplemented by two secondary storage systems: the phonological loop, which is dependent on sound and language, and the visuospatial sketchpad, which contains visual information. The phonological loop is hypothesized to comprise two sub-components: a phonological store and an articulatory control process based on inner speech. The central executive is the model’s most significant component. It is considered the seat of attentional control and is in charge of monitoring, regulating, and adapting memory processes. Baddeley [32] further developed the theory by adding a new component of working memory termed episodic buffer, which is capable of binding information from subsidiary systems and long-term memory into a unified episodic representation. The primary route of retrieval from the buffer is thought to be conscious awareness.

Tulving [33] focused their research interest on long-term memory (LTM). According to this model, LTM is comprised of three distinct but interactive subsystems, namely semantic, episodic, and procedural memory. In brief, semantic memory organizes meanings. Episodic memory stores events and procedural memory is the part of memory that is responsible for skill acquisition.

Craik and Lockhart [34] introduced an alternative framework that recognizes memorization as the outcome of the depth of memory processing. According to their research, stimuli are processed on multiple levels which in turn determine the strength as well as the durability of the memory traces. In other words, the deeper levels of semantic or cognitive analysis are, the longer-lasting, and stronger memories will be. The depth of processing plays a crucial role in coding and retention, as well as retrieval of memories. The researchers supported the idea that, although processing is unconscious, individuals with the power of their attention as well as the use of strategies can take control over processing by using, for instance, vivid visual images or creating strong associations between previous and new memories. In addition, they regulate the depth of processing by utilizing, for instance, maintenance rehearsal for temporary retention or elaborative rehearsal for deeper consolidation.

Other researchers enriched memory theory with important observations and findings regarding the possible strengths and limitations of human memory. Miller, for instance, supported the hypothesis that working memory has a limited span, which is generally seven items [35]. The limited memory span provides a possible explanation for humans’ difficulty in effectively recalling strings of letters or numbers. As a result, researchers concluded that learners are required to use techniques for organizing information into smaller manageable units.

Time and durability constraints have been also described. Ebbinghaus’s Forgetting Curve is a visual representation of the way memories fade over time. According to this model, forgetting is inevitable and takes place at a certain rate, unless learners employ strategies to prevent the tendency for the deterioration of their memories [36,37].

The term metamemory was introduced by Flavell to describe the knowledge of and control of the memory processes. Flavell describes metamemory as a form of “intelligent structuring and storage of input, intelligent search, and retrieval operations, and intelligent monitoring and knowledge of these storage and retrieval operations” [38].

Nelson and Narens [39] developed an innovative theoretical metamemory framework whose basic hierarchical structure consists of two interrelated levels termed the object-level and the meta-level. This metamemory model is under the rule of metacognitive monitoring and control processes in the stages of acquisition, retention, and retrieval of memory contents.

### 3.2. Factors Affecting Memory and Metamemory Abilities

Circadian rhythms refer to the regular cycling of biological phenomena that regulate various neurophysiological events such as synaptic plasticity and hormonal regulation. Recent studies reveal that time-of-day effects and circadian rhythms have a major impact on memory formation [40].

Sleep, too, has a positive impact on memory processes, boosting consolidation processes and making memories accessible and recognizable. Especially post-learning sleep can significantly help learners to recall previous knowledge [41].

Brain waves, the oscillatory rhythms of the brain, which determine the functioning of higher mental abilities, have been found to play an important role in memory control processes, modulating the brain activity of brain regions such as the hippocampus and prefrontal cortex [42].

Dietary factors play an important role in maintaining memory in a good condition. People who are aware of the functionality of memory consume brain foods to boost memory performance. Vitamins C, vitamin D, B vitamins, carotenoids, selenium, Omega-3 fatty acids, minerals, and the use of supplements comprise a short list of the nutrients that are necessary for healthy memory functions [43]. Blood glucose levels affect the functioning of memory. Thus, glucose regulation by avoiding sugar is necessary [44].

Regulation of the oxygen–carbon dioxide ratio is considered an important factor for memory function. Therefore, breathing exercises help improve memory [45].

Physical exercise is closely associated with memory functions. Exercise training is considered a well-recognized intervention for people with memory impairments. Exercise training enhances memory flexibility via neuroplastic alterations, neuroprotective effects, and neurotrophic factors signaling. Exercise also regulates oxygen levels and blood glucose levels, which play a crucial role in memory [46].

Psychological anxiety and the stress hormones/neurotransmitters released during and after stressful situations slow down memory processes and inevitably lead to academic underachievement [47]. Heavy stress holds metacognitive abilities back, resulting in an inability to monitor and control memory processes. On the contrary, relaxation techniques help learners to take conscious control of cognition including memory meta-abilities [48].

## 4. A Metamemory Model Based on Metacognition Principles

Theories of memory and metamemory directly or indirectly recognize that the functions of memory are to some extent under human control through the employment of strategic interventions. It is also recognized that metacognition is closely linked to the concept of metamemory. In the following section, we present a metamemory framework based on metacognition. This model integrates the existing knowledge of metamemory and metacognition into eight distinct but complementary components [13,14,29,30,31,32,33,34,35,36,37,38,39].

### 4.1. Metamemory Knowledge

Metamemory knowledge represents a set of meta-skills enabling one to acquire explicit knowledge and understanding about (a) how memory operates, (b) how metamemory abilities gradually develop, and (c) how metamemory strategies can be applied [13,14]. The first metamemory component also includes knowledge acquisition about memory’s capacities, limitations, and idiosyncrasies. The knowledge of metamemory strategies as well as the processes involved in memory monitoring and control are also considered important variables [49,50]. Metamemory knowledge requires systematic instruction. According to Karably et al. [51], students show better strategy use and more strategy transfer when explicit metamemory instructions are provided.

### 4.2. Applied Metamemory

Applied metamemory refers to a set of awareness skills arising from experience, action, and practice through which learners can objectively evaluate memory-related strengths, weaknesses, opportunities, and threats [13,14]. The mediation of experience, practice, and maturation ensure that one can strategically apply metamemory knowledge in encoding or retrieval processes considering the memory-relevant variables that affect their performance in a given task [49]. Metacognitive experiences include awareness of external and internal constraints. External constraints concern parameters such as task variables, while internal constraints refer to personal variables such as cognitive status, expectations, and personal beliefs [13,14]. To meet the task objectives, one should be experienced in the applicability of metamemory strategies and techniques. For instance, studies have already shown that younger students are less able to decide what the best metamemory strategies are in each memory-relevant situation. The ability to apply metamemory knowledge presupposes reflective thinking skills to self-evaluate memory strengths and weaknesses [52]. Young and healthy students tend to overestimate or underestimate their memory abilities, while significant difficulties are also observed in children with learning disabilities [51,53]. Researchers also hypothesize that patients with memory disorders suffer from a reflective memory awareness process, which results in a state of unawareness of memory difficulties and consequently inability to plan, predict, and voluntarily apply metamemory strategies [52].

### 4.3. Metamemory Observations

An essential component of the metamemory framework is considered the meta-skill of online monitoring of the memory contents, the progress of the mnemonic information processing, and the use of metamemory strategies. It is a form of introspection and conscious control governed by attentional control processes at different stages of memory processing. It involves the meta-process through which learners consciously turn attention inward to shed light on the contents of memory during the stages of encoding, retention, or retrieval [13,14,54]. Metamemory observation presupposes the simultaneous fulfillment of at least two basic parameters, namely, the ability to perceive the memory phenomena along with the real-time observation of this perception [50]. In other words, it is a meta-perception process. Systematic observation can help learners to identify errors during encoding or retrieval, identify “difficult” information, facilitate memory organization, and even recognize forgetfulness and mind wandering [55].

### 4.4. Metamemory Self-Regulation

Although a large part of memory operates on an unconscious level, there is another part that is conscious, observable, and under human control. Memory control refers to a set of metastrategic regulations during the stages of encoding, maintenance, and retrieval by which learners can manage, for instance, information availability in working memory, inhibit memories, and make decisions about the accessibility of information in the memory system [13,14,56]. It is worth noting that recent studies reveal that subconscious training strategies can also improve memory regulation [57].

### 4.5. Metamemory Adaptability

Adaptability is considered an inherent characteristic of memory. Mental flexibility, for instance, allows working memory to act as a workspace on which information can be held, manipulated, and used to guide behavior [58]. Studies have shown that limited adaptive skills constitute a predictor for memory problems [59]. A well-developed metamemory requires learners to be skilled to approach difficult tasks flexibly using metamemory knowledge and utilizing principles of memory to adjust learning strategies in novel contexts [60,61]. Gifted students stand out from their peers because they are flexible, creative, and insightful enough to devise new memory strategies and apply them in transfer tasks [61]. Metamemory flexibility constitutes a factor of health balance encouraging attention to shift from negative memories to positive ones [62].

### 4.6. Recognition Metamemory

Recognition refers to the ability to accurately identify a recently encountered item as having been presented previously. Recognition, for instance, involves recognizing and recalling someone’s name by seeing his/her image [63]. Each time an event is reexperienced, one is called upon to match this content to stored memory representations [64]. Metamemory recognition skills enable individuals to identify similarities, seek new routes to recollection, and recognize memory errors [13,14,15,63].

### 4.7. Metamemory Discrimination

Metamemory discrimination describes the set of filtering meta-abilities that permit learners to make accurate judgments and, in turn, appropriate memory-related decisions. For instance, each time learners are bombarded with information, they should be able to apply filtering rules, selecting the most important information according to the circumstances [13,14]. Judgments concern a wide range of memory-related situations. Students, for instance, often make inaccurate judgments about their ability to recall new information. Metamemory judgments enable the learner to distinguish diverse bits of information, true from false beliefs, and to deal with memory bias (i.e., recall bias) [65,66]. Accurate judgments during learning new material can lead to successful decisions and strategy selection. For instance, students can easier make decisions about the allocation of study time [39]. Inaccurate metamemory judgments may be closely linked not only to academic under-achievement but also to psychiatric symptoms such as obsessive and compulsive behaviors since it is often observed limited capacity to evaluate the truthiness of memories [67].

### 4.8. Mnemosyne

**Figure 1:**
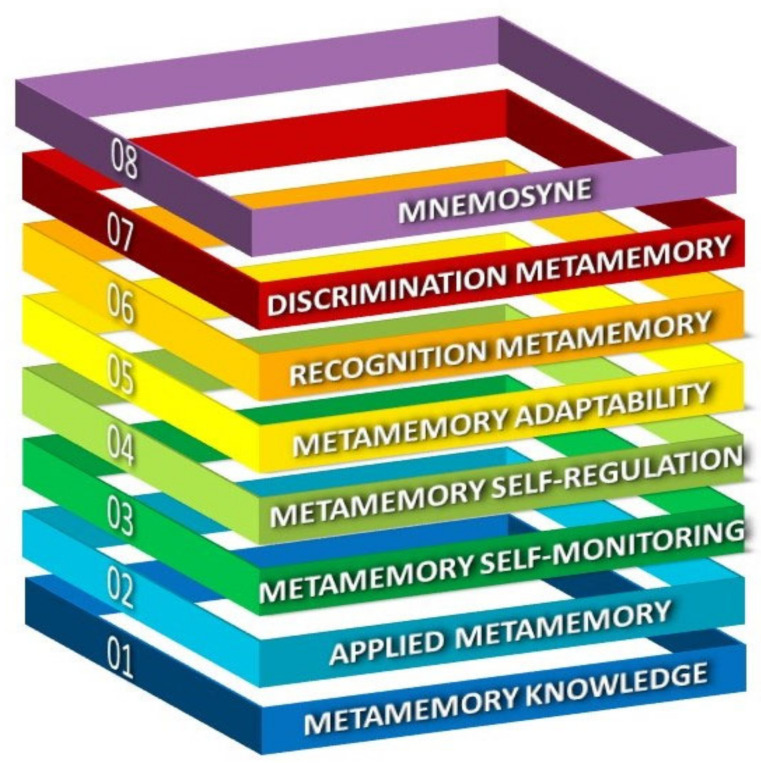
The eight pillars of the metamemory model based on metacognition. The metamemory model consists of eight distinct but complementary components.

Mnemosyne refers to a state of pure awareness that upholds memories accessible to remind individuals of their self-identity [13,14,68]. It is not accidental that people with severe memory problems, along with the reduced awareness of metamemory abilities, experience a loss of self-knowledge and an inability to update the representation of their self [69]. Reductions in such self-related meta-abilities have a major impact on decision-making capacity. The ability to “remember to remember” is closely associated with self-knowledge and awareness. When an individual is aware that their memory is impaired, then they are more likely to apply metamemory strategies that compensate for memory problems [13,14,70] (Figure 1).

## 5. Metamemory: Metacognitive Training Strategies

The ability to voluntarily recall information and self-manage memory functions is considered a crucial factor in academic achievement, psychological health, and well-being. As an individual progresses to higher and more complicated levels of thinking, the capacity to regulate memory becomes increasingly challenging. Researchers believe that the use of strategies with the aid or not of assistive technologies can help individuals to overcome memory constraints by taking control of memory operations [9,10,11]. In the first subsection, we present a set of evidence-based strategies, while the second subsection investigates the role of virtual reality and mobiles as assistive tools in metamemory training [22,23,24,25,26,27,28].

### 5.1. Metacognitive Training Strategies

The Method of Loci is an ancient mnemonic strategy used to enhance recall. It is performed by mental navigation to a familiar environment, ‘placing’ the elements to be remembered in specific locations. This technique impels learners to link new information to previous knowledge, organize information, and create associations. In addition, it requires intense attention and observation [10]. Wagner et al. [71] used the method of loci as a training intervention and found that consolidation and recall abilities were significantly improved. The speed of transferring information from working memory to long-term memory increased, and the durability of memories was strengthened. It is noteworthy that control skills, attention, and organizational flexibility were also improved. At the neuronal level, an enhancement of the connectivity between the neocortex and the hippocampus was observed, which indicated better consolidation.

A mind map constitutes a diagram that includes a central image, main themes projecting from the central image, branches with key images and keywords, plus branches forming a linked nodal structure [72]. The mind mapping technique requires the learner to recognize important topics and to organize and filter information. Kalyanasundaram et al. [72] evaluated the effectiveness of the mind-mapping technique in information retrieval among 64 medical college students. The results showed that the mind-mapping technique improved information retrieval.

The dual-coding technique depends on the theory developed by Paivio (1971), according to which memory for verbal information is strengthened if corresponding imaginal information is activated, and such engagement of both verbal and nonverbal systems results in the dual coding of information, making memories powerful [73].

Reading aloud is another metamemory technique that is based on the hypothesis that the simultaneous activity of speaking and hearing oneself can boost filtering and better organization of information into long-term memory. Reading aloud also requires active engagement, awareness, and self-referential auditory input [74].

Linking and chunking strategies allow the learner to organize and flexibly manage information according to their memory strengths and limits [75]. According to Norris et al. [75], chunking permits the information capacity of short-term memory to be exploited more efficiently. In addition, it facilitates learners to retrieve information, such as lists of numbers.

The principle “use it or lose it” is based on the hypothesis that memories are doomed to be lost unless systematic repetition intervenes. Indeed, research has shown that systematic repetition of the already learned information at regular intervals activates and re-organizes brain areas that play a critical role in fast and effortless recall [76].

Rehearsal refers to the conscious repetition of information which intends to prolong the maintenance of the information assuring that the information will pass on to the next level of processing. However, it is considered a less powerful strategy compared with visual strategies [77].

Elaboration enables the learner to achieve a deeper consolidation of memories. Elaboration strategies train learners to connect previously learned with new information. Using analogies, previous experiences, or explaining the concepts to be learned, learners can also create strong memories with episodic and semantic features [55].

Self-testing methods are considered metacognitive and retrieval-based strategies. Self-testing is the process of evaluating the accuracy of how well one can remember previous knowledge. According to Rodriguez et al. [78], self-testing not only makes memories more durable, but also trains learners’ metamemory abilities. Specifically, learners pose self-questions, select and apply memory strategies, and make self-evaluations about what they remember and what they do not remember. In addition, this retrieval-based strategy depends on self-monitoring skills. Self-testing combined with spaced repetition has been found to maximize memory performance and academic achievement [78].

Learning by teaching method constitutes a conscious practice of retrieval and knowledge re-organization. Applying this method, learners “re-download” and reprocess previous knowledge on a much deeper level [79,80]. Nestojko et al. [80] asked students to study passages either in preparation for a later examination or for teaching the passage to another student. The findings revealed that students who planned to teach produced more well-organized free recall of the material. In addition, they more effectively used metacognitive learning strategies to facilitate recall.

Attention training techniques (e.g., meditation) strengthen attention, reduce cognitive load, increase memory resource availability, make memory more flexible, and strengthen visuospatial skills [68,81].

Context-based training plays a crucial role in filtering information. Considering the context during memory practice, learners can facilitate memory encoding and storage [55]. Sensory stimulation techniques can keep memories “alive” by enhancing both the process of encoding and recall. Multisensory learning could significantly contribute to this goal [55]. Emotions play a crucial role in encoding, consolidation, and retrieval processes. Adding emotional content to the information to be learned can help memorization [55]. It is no coincidence that the same structures involved in the processing of memories are also involved in the processing of emotions [82].

Other effective strategies include the use of acronyms, acrostics, story-based methods, the keyword method, the snapshot technique, and the double keyword method [11,83].

Hypnosis techniques can help people to improve their memory abilities. Lindeløv et al. [57], using a randomized controlled trial, found that hypnotic suggestions restored working memory performance in patients with memory impairments. Hypnotic techniques can also achieve memory restructuring, activating previous unhelpful memories, modifying them through hypnotic suggestions, and reconsolidating them. Hypnosis techniques can also help people to retrieve forgotten but not lost memories [84,85,86].

Cues are stimuli that help subjects to recall memories. Recent studies reveal that the use of subliminal training strategies with the use of subliminal cues, symbols, and images can improve memory performance. Specifically, subliminal strategies are associated with the better recall, improved short-term memory, better metamemory skills, and memory self-efficacy. Subliminal cues can facilitate retrieval of information, reduce cognitive load, and help working memory operate effectively. In addition, the use of subliminal positive messages elevated positive mood and motivation boosting memory performance [87].

Neurolinguistic programming (NLP) is a psychological approach that utilizes methods and techniques for memory restructuring. NLP and positive psychology share a common ground in strategies use [88]. NLP strategies include positive affirmations, positive visualizations, imitation techniques, anchoring, reframing techniques, and storytelling methods. NLP strategies have the potential to alter unhelpful memories or unhelpful beliefs about metamemory abilities. They can also motivate, relax, and enhance positivity, boosting memory control meta-abilities [88,89].

### 5.2. Virtual Reality and Mobile-Assisted Metamemory Training

Krokos et al. [90] applied the spatial mnemonic technique known as the memory palace in a virtual environment. The main objective of the study was to compare the efficacy of the memory palace either in an immersive virtual reality condition or in a traditional desktop condition. The results showed that the sample of 49 participants had better spatial awareness and could better recall information in the virtual reality condition compared with the control group.

Vindenes et al. [91] designed “Mnemosyne”, an immersive virtual reality memory palace to improve recollection abilities in a sample of eighteen participants. The application enabled the users to create personalized memory palaces by exploring and collecting “memory cubes” in a virtual environment via a head-mounted display. The results of the pilot study revealed positive effects on recall abilities. The researchers also observed that the virtual memory palace was more beneficial for participants with higher spatial reasoning abilities.

Wais et al. [66] investigated whether a virtual reality spatial wayfinding game could improve long-term capabilities as well as mnemonic discrimination abilities. A total of forty-eight participants were randomly assigned to the following three conditions: computer games, Labyrinth-VR games, or placebo control games. The VR game presented engaging, adaptively increasing challenges to explore virtual urban and village communities and then navigate across them to fulfill given errands. The results revealed post-treatment improvements in high-fidelity long-term capability. In addition, participants in the VR group showed better flexibility to differentiate between diverse bits of information.

Optale et al. [22] conducted a randomized controlled trial to investigate the effectiveness of a virtual reality memory monitoring intervention in memory control. The immersive VR memory training intervention included auditory stimulation and VR experiences in pathfinding. The control group underwent face-to-face training sessions using music therapy. The VR memory group significantly improved in memory tests, particularly in long-term recall. The authors concluded that virtual reality memory training had positive effects for the additional reason that virtual reality improves attention abilities.

Man et al. [92] investigated the effectiveness of non-immersive VR-based memory training. Forty-four participants were randomly assigned to a VR-based and a therapist-led memory training group, respectively. Both groups showed positive training effects, with the VR group improving objective memory performance more than the non-VR group.

Gabana et al. [93] explored the effectiveness of a VR memory training game on working memory performance in a sample of thirty participants. A custom video game for desktop and VR with three difficulty levels was created to evoke distinct levels of arousal while maintaining the same memory load for each difficulty level. The results showed that participants, especially those with low working memory capacity, performed better in VR game conditions. Immersion in a VR game helped users to make good use of their cognitive resources and remain activated and motivated. The researchers concluded that virtual reality enhanced working memory for the additional reason: Virtual reality can easier modulate users’ affective state (i.e., evoking different levels of arousal and positive valence) inducing a state of flow, which in turn enhance memory performance.

Research has shown that extinction applied after the retrieval of a craving-related memory may inhibit reconsolidation and thus prevent craving. Liu et al. [94] presented a study protocol for the implementation of memory retrieval and extinction training in virtual reality. The study aimed to examine the efficacy of virtual reality in dealing with drug-associated memories. Zandonai et al. [95] investigated whether virtual reality can reactivate, extinct, and prevent the reconsolidation of craving memories. The results showed that virtual reality memory manipulation can help smokers regulate memories that trigger cigarette cravings.

Sandberg et al. [24] designed and evaluated the effectiveness of a Method of Loci training program in a smartphone application. A total of 359 adults played a memory training game which consisted of a sequence memory task and instructions to use the Method of Loci for encoding and retrieval for three months. The users completed memory tests on different occasions during training and answered brief questionnaires, all within the app. The results showed that mnemonic training in a smartphone application improved participants’ memory ability.

Oh et al. [25] investigated whether an 8-week smartphone-based memory reinforcement training could enhance memory performance. The application was designed to train attention along with working memory, because of their bi-directional relationship. Fifty-three adults were randomized into either one of two intervention groups (mobile memory training group, Fit Brains^®^) or a wait-list group. The results showed that the mobile-based memory training intervention significantly improved participants’ working memory capacity.

Hermes et al. [96] conducted a randomized controlled pilot study to assess the effectiveness of a mobile application for training visual mnemonic techniques. A total of 14 participants were divided into the experimental and the control group. After a four-week training, participants in the mnemonic app group showed a slight improvement on standard memory tests.

Scullin et al. [97] conducted a four-week randomized controlled trial to explore whether smartphone-based strategies can improve prospective memory in fifty-two participants with mild cognitive impairment. The training included the use of a reminder app or digital recorder app. The results showed that mobile memory aid can reduce forgetting events, improve prospective memory, and facilitate independent daily functioning.

El Haj et al. [98] investigated the efficacy of smartphone calendars on prospective memory in Alzheimer’s disease. Twenty-two participants were divided into two groups. The experimental group was cued with a smartphone calendar application, while the control group was with a paper-and-pencil calendar. It was revealed that smartphone intervention resulted in improved monitoring skills and reduced omissions of prospective events. The researchers outlined that the smartphone group outperformed because smartphone intervention reduced cognitive load and elevated motivation.

Hackett et al. [99] investigated the efficacy of a smartphone reminder on a sample of ten participants with mild cognitive impairment or mild memory disorder. The smartphone generated alert sounds with an auditory alarm at a prescribed period to direct the user to the phone. Brief messages helped participants to complete tasks. The results showed that smartphone has a significant potential to reduce functional disabilities derived from problems in memory monitoring, planning, and organization skills.

Visser et al. [100] investigated the effects of smartphone-based autobiographical memory training on memory bias and memory modification. The training was designed to familiarize participants with the process of retrieving autobiographical memories with either a positive, neutral, or negative emotional valence. One-hundred and fifty-three participants were randomly divided into the positive, negative, or neutral memory condition. The retrieval of autobiographical experiences was repeatedly prompted through the mobile application. Participants in the positive group were asked to retrieve positive events, whereas participants in the negative condition were instructed to retrieve negative memories. The results showed positive training increased positive memory bias, improved retrieval of positive memories, and lowered depressive scores.

## 6. Discussion

Although learners can be strategic and do possess some metamemory skills, they tend to be somewhat less skilled at comprehending the various influences on memory and at applying metacognitive strategies for facilitating memory operations. In addition, teachers frequently confront difficulties in teaching students the information and skills needed for success, because they are not always well-trained on how to help students learn how to remember [51]. Thus, it is apparent that effective teaching requires systematic metamemory training through instruction and practice to help students develop their metamemory skills and strategies and, thus, become self-regulated learners.

Virtual reality provides a wide range of opportunities for memory and metamemory training. The possible reasons why virtual reality can help people to improve memory control are the following. Virtual reality provides an entirely controlled environment. This implies that stimuli can be adapted to the participant’s needs. Specifically, sensory input can be decreased to keep the cognitive load down or can be increased to stimulate memories by providing a multisensory environment. Virtual reality excels compared with other technologies in that it can manipulate attention and boost attentional control functions. Distractions are easier to eliminate in virtual reality, allowing the working memory to operate flexibly and the learner to take the appropriate metamemory decisions. VR also depends on visualization through which the ability to store and recall memories is enhanced. It can also be helpful in cases of problems with verbal memory. In virtual reality, it is easier to control arousal levels and mobilize those positive emotions that facilitate better control of memories, especially in cases where virtual reality and games coexist. In addition, virtual reality can facilitate autobiographic memory retrieval, which provides great opportunities for people with memory impairment. Most important, VR facilitates the reactivation, extinction, and reconsolidation of memories boosting relearning and unlearning processes [93,101,102]. Therefore, it is apparent that virtual reality provides a wide range of opportunities for metamemory training that permit learners to take control of their memory, make appropriate decisions, and acquire a better understanding of memory functions.

Mobile technologies, cloud-based platforms, deep learning-driven software algorithms, and automated physiological sensors are promising tools for memory rehabilitation. Mobiles provide cost-effective memory training solutions, are easily accessible to a wide range of people, and do not stigmatize patients with memory difficulties [98].

However, some researchers are cautious about the effectiveness of virtual reality and smartphones in metamemory training. For instance, virtual reality alone does not guarantee better memory performance. Several factors may play a role in the effectiveness of the intervention, such as the users’ characteristics and the design of the environment. In addition, users often report nausea, headaches, and eyestrain due to a large number of visual stimuli in virtual reality environments. Researchers also outline that smartphone overuse and overdependence may lead to diminished metamemory abilities [103,104,105].

It is worth noting that the inclusion of digital devices in memory training interventions requires the cultivation of a positive attitude toward using assistive technologies. Professionals, therapists, and educators should be aware of the benefits and the special features of mobiles and virtual reality to facilitate their memory training programs and help students to be familiarized with the use of memory technologies [106].

Our study raises several opportunities for future research regarding the evolution of the metamemory theory as well as the investigation of the effectiveness of metamemory strategies within digital environments. The proposed metamemory strategies framework supported by virtual reality and mobile applications provides a training paradigm that can be applied with appropriate adjustments in different contexts and populations.

## 7. Conclusions

The current study aimed to develop a metamemory model based on the principles of metacognition. The theoretical model was supported by evidence-based metacognitive strategies for memory control. Finally, special emphasis was given to the role of mobiles and virtual reality as assistive tools in metamemory training.

We conclude our investigation with the following observations. Memory, to a significant extent, is trainable and under human control. The use of metacognition-based training strategies enables learners to develop metamemory skills and abilities. Learners, through metacognition-based memory training, can be in readiness to manipulate memory operations. Most important, metamemory training aims to develop memory awareness skills. Even the unconscious part of the memory can be trained to some extent, provided that metamemory knowledge exists along with appropriate strategies use. In this study, we described a wide range of memory-control strategies in the light of metacognition. These strategies have the potential to train both conscious as well as non-conscious parts of the memory. Thus, the use of metacognitive strategies not only guarantees metamemory improvements, but also paves the way for the development of exceptional memory skills.

Digital devices, such as mobile applications and virtual reality, were found to be promising technologies for the implementation of memory-control strategies assisting in various ways. We conclude that the integration of digital technologies is quite effective and rewarding and promotes educational procedures through the use of mobile devices, different ICTs applications, and games. In addition, the blending of ICTs with metacognition theories and models, mindfulness, meditation, and emotional intelligence theories as well as with the knowledge about the role of environmental factors and nutrition advances and boosts memory abilities [14,40,41,42,43,44,45,46,47,48,68,81,84,85,88,89,90,91,92,93,94,95,96,97,98,99,100].

More research is needed with large-scale experimental studies testing the efficacy of the metamemory strategies framework, especially in clinical populations. Additional study on assistive technologies in metamemory skills training is required. Finally, research on the efficacy of metamemory strategies with or without the use of technologies in school or therapeutic settings is critical.

## Data Availability

This study did not report any data.
